# Cryptochromes and Hormone Signal Transduction under Near-Zero Magnetic Fields: New Clues to Magnetic Field Effects in a Rice Planthopper

**DOI:** 10.1371/journal.pone.0132966

**Published:** 2015-07-14

**Authors:** Gui-Jun Wan, Wen-Jing Wang, Jing-Jing Xu, Quan-Feng Yang, Ming-Jiang Dai, Feng-Jiao Zhang, Gregory A. Sword, Wei-Dong Pan, Fa-Jun Chen

**Affiliations:** 1 Department of Entomology, Nanjing Agricultural University, Nanjing, Jiangsu, China; 2 Beijing Key Laboratory of Bioelectromagetics, Institute of Electrical Engineering, Chinese Academy of Sciences, Beijing, China; 3 Department of Entomology, Texas A&M University, College Station, TX, United States of America; Syddansk Universitet, DENMARK

## Abstract

Although there are considerable reports of magnetic field effects (MFE) on organisms, very little is known so far about the MFE-related signal transduction pathways. Here we establish a manipulative near-zero magnetic field (NZMF) to investigate the potential signal transduction pathways involved in MFE. We show that exposure of migratory white-backed planthopper, *Sogatella furcifera*, to the NZMF results in delayed egg and nymphal development, increased frequency of brachypterous females, and reduced longevity of macropterous female adults. To understand the changes in gene expression underlying these phenotypes, we examined the temporal patterns of gene expression of (i) *CRY1* and *CRY2* as putative magnetosensors, (ii) *JHAMT*, *FAMeT* and *JHEH* in the juvenile hormone pathway, (iii) *CYP307A1 *in the ecdysone pathway, and (iv) reproduction-related *Vitellogenin *(*Vg*). The significantly altered gene expression of *CRY1* and *CRY2* under the NZMF suggest their developmental stage-specific patterns and potential upstream location in magnetic response. Gene expression patterns of *JHAMT*, *JHEH* and *CYP307A1* were consistent with the NZMF-triggered delay in nymphal development, higher proportion of brachypterous female adults, and the shortened longevity of macropterous female adults, which show feasible links between hormone signal transduction and phenotypic MFE. By conducting manipulative NZMF experiments, our study suggests an important role of the geomagnetic field (GMF) in modulating development and physiology of insects, provides new insights into the complexity of MFE-magnetosensitivity interactions, and represents an initial but crucial step forward in understanding the molecular basis of cryptochromes and hormone signal transduction involved in MFE.

## Introduction

Magnetoreception or magnetosensitivity, the ability of living organisms to respond to magnetic environments such as the geomagnetic field (GMF) and various artificial magnetic fields, has been well documented, especially in the animal kingdom [1, 2]. Much work has been done in relation to animal magnetic orientation in birds, fish, turtles, lobsters and even insects [3, 4]. At present, there are two non-mutually exclusive models of magentoreception, the light-independent magnetite-based system and the light-dependent radical pair mechanism (RPM). Both models have received experimental and theoretical support [3]. In particular, the putative RPM occurring in the flavoprotein photoreceptor cryptochromes (e.g. RPM with FAD–Trp, or FAD-Z) has received much more attention [5–8]. Cryptochromes can be grouped into three classes including animal cryptochromes (Type I, Type II and Type I+II), plant cryptochromes, and CRY-DASH cryptochromes [9]. Despite this seeming diversity, cryptochromes are fundamentally similar in both structure and photochemistry enabling them to have the potential to detect not only light, but also redox state and geomagnetic field [10]. The similar characteristics in different types of cryptochromes may also be the reason why animals that don’t migrate retain the capacity for magnetosensitivity, e.g. the honeybee *Apis mellifer*a [11], the fruit fly *Drosophila melanogaster* [6]. Moreover, using a transgenic approach, human CRY2 was proved to even function as a magnetosensor in the magnetoreception system of *Drosophila* [12]. Thus, cryptochrome is likely to be a conserved and common magnetosensor of animals in a light-dependent manner, that is not limited to use in orientation.

Marley *et al*. (2014) advanced our understanding of the neuronal signal transduction process involved in the immediate electrophysiological magnetosensitivity by demonstrating a cryptochrome-dependent magnetic field effect on seizure response in *Drosophila melanogaster* larvae. And the data is consistent with a magnetosensitive, photochemical radical pair reaction in cryptochrome that alters levels of neuronal excitation [13]. It is commonly known that hormone signal transduction conventionally coexists and works together with neuronal signal transduction. The juvenile hormone (JH) and the principal molting hormone (MH) (i.e. ecdysone), 20-hydroxy-ecdysone (20E) are highly versatile insect hormones that coordinate development, growth and reproduction [14, 15], and signaling by these two hormones is regulated by neuropeptides and environmental signals [14–19]. By analogy, we hypothesized that the phenotypic MFE on development and physiology as we observed in small brown planthopper, *Laodelphax striatellus* and brown planthopper, *Nilaparvata lugens* subjected to a near-zero magnetic field (NZMF) [2], may be associated with cryptochrome-related hormone signal transduction which would alter the secretion of JH and ecdysone. Actually, NZMF has also been reported to affect circadian rhythm [20, 21]. And it is generally accepted that cryptochromes are not only the putative primary magnetosensors, but also key components in regulating circadian rhythms, which are ultimately involved in controlling the downstream expression of specific hormones in both vertebrates [22] and invertebrates [23, 24].

To investigate the hypothesis, we used the white-backed planthopper, *Sogatella furcifera*, which has two cryptochromes, CRY1 and CRY2. This insect is a major migratory insect pest of rice crops, causing serious loss by sap-sucking and transmitting the southern rice black-streaked dwarf virus [25]. Adult females of *S*. *furcifera* exhibit wing dimorphism and occur in two forms, macropterous with functional wings and brachypterous with reduced wings. Males are usually monomorphic macropterous [26]. Previously, we have found the phenotypic MFE on development and physiology of the two other species of rice planthoppers when subjected to a NZMF [2]. This is also the first reason we chose the NZMF as the experimental magnetic environment in this experiment, which ensure the feasibility of the NZMF in inducing MFE on rice planthoppers. Besides, there are two more practical advantages. First, GMF decay is a real phenomenon. A recent snapshot of the GMF based on data from the ESA’s Swarm satellite indicates a GMF decay 10 times faster than expected [27]. Moreover, the current trend of changing GMF looks persistent [28]. NZMF is a good approximation to simulate GMF decay. Ongoing decay in GMF provide a unique opportunity to study its potential bioeffects on organisms as well as role of field intensity in magnetoreception and life evolution [3, 29, 30]. Second, the strength of the galactic magnetic field does not exceed 0.1 nT, and interplanetary navigation will expose life to magnetic environments near 1 nT that are well below the typical value of approx. 0.05mT at the earth’s surface [30]. Spaceflight missions are proved to impact circadian clocks and disrupt sleep which may be due to the absence of electromagnetic field in space [31, 32]. Therefore, NZMF is also regarded as a good simulator for the magnetic environment in space, and it would help a lot in exploring the mechanisms of the adverse effects in a MFE context.

To date, no mechanisms have been identified whether hormone signal transduction is a part of a GMF- or artificial SMF-induced phenotypic response. To explore the potential hormone signal transduction and its link to cryptochrome in a molecular context, we exposed *S*. *furcifera* to a manipulative NZMF and quantified the temporal patterns of gene expression of the putative magnetosensors, *CRY1* and *CRY2*, *JHAMT*, *FAMeT* and *JHEH* in JH pathway, *CYP307A1* in ecdysone pathway, and *Vitellogenin* (*Vg*) which is downstream the JH hormone pathway [33] and crucial for fecundity [34].

## Materials and Methods

### Ethics statement

No specific permission was required for the collection location of *Sogatella furcifera*, a rice planthopper, and no endangered or protected species were involved, all experiments were done in controlled laboratory conditions.

### Insect rearing

The white-backed planthopper, *Sogatella furcifera*, was collected from the paddy fields of Jiangsu Academy of Agricultural Science at Nanjing, Jiangsu Province of China. The planthopper stocks were reared on rice seedlings (cv. TN1; 15–30 days after planting, DAP) grown in the same environment chambers (HPG280H, Ningbo JIANGNAN Ltd., Ningbo, China) at 70–80% RH, 27±1°C/26±1°C day/night temperatures, and 14:10h (L:D) photoperiod for twelve generations to obtain uniform colonies. The brachypterous wing forms were only found in female adults, as previously reported [26]. Considering that macropterous females are of considerable significance as pests due to their migratory ability and potential magnetic sensitivity for orientation. we used macropterous females to measure fecundity, longevity and the expression patterns of selected genes.

### Setup of magnetic field and insect exposure treatments

**Fig 1:**
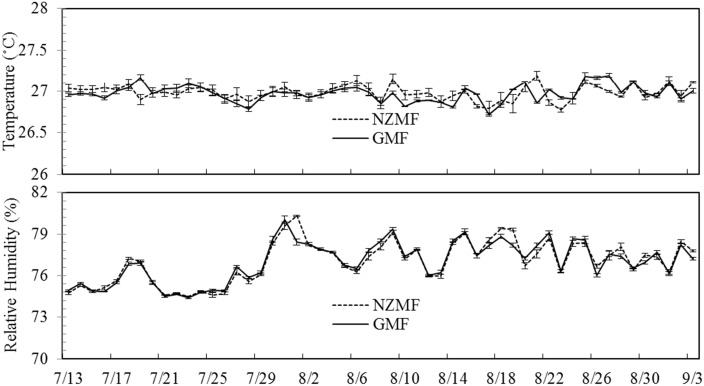
The dynamics of temperature and relative humidity (RH) in near-zero magnetic field (NZMF) vs. geomagnetic field (GMF) treatments from 13 July to 3 September 2014. Significant differences in temperature or RH between NZMF and GMF treatments during the egg, nymphal, adult and total developmental period of the white-backed planthopper, *Sogatella furcifera* were respectively tested for using paired t-tests with an alpha value of *P*<0.05.

A static near-zero static magnetic field (NZMF) was generated by four pairs of Helmholtz coils mounted in a laboratory with an average intensity of ~500nT measured using a fluxgate magnetometer (CTM-5W01B, National Institute of Metrology, China, sensitivity: ±1 nT) in the center cubical space (300 × 300 × 300 mm^3^) as described in our previous work [2]. An identical set of equipment was used as control to simulate the real geomagnetic field, and both sets of equipment were interchanged during the experiment to minimize variation due to equipment. As described in Wan *et al*. (2014), the magnetic field equipment for both treatments were each housed in customized cylindrical chambers to maintain constant lighting and environmental factors. The experimental *S*. *furcifera* treatment groups were both reared on 15–30DAP rice seedlings (cv. TN1) in glass tubes (diameter: height = 3.0cm: 15cm), and exposed to either a NZMF or simulated GMF. The rice seedlings were exchanged every 2 days and randomly arranged within the NZMF or GMF treatments to minimize the coils' fringe field effects. Each glass tube was covered with a piece of 40-mesh nylon gauze to prevent the escape of planthoppers. Kimura B culture solution was added along the tube wall daily to provide sufficient water and nutrients for the seedlings. The magnetic flux density of the NZMF and GMF was measured daily. The NZMF and GMF treatments were located at the same position within the effective magnetic field area to ensure that illumination intensity, temperature, humidity, vibrations and disturbances potentially induced by experimenters were as uniform as possible. The experiment was conducted under a 14:10 h (light: dark) photoperiod and monitored continuously using an automatic temperature analysis system (U23-001, HOBO Pro V2 Temp/RH Data Logger, MicroDAQ.com, Ltd., Contoocook, NH, USA) with an accuracy of ±0.02°C from 0 to 50°C. No significant differences in temperature (paired t test, *P*≥0.11, n = 53) or RH (paired t test, *P*≥0.13, n = 53) between NZMF and the GMF during the period from egg to adult respectively were found (Fig 1).

### Developmental period of eggs and nymphs

Thirty female and male pairs of newly emerged macropterous *S*. *furcifera* were randomly selected from the insect stocks, and separately reared and mated in pairs on rice seedlings within glass tubes for 2 days in a greenhouse. Each mated female was then transferred into either NZMF or GMF treatments in the same cage to oviposit on fresh rice seedlings for one day, and then all adults were removed. Cages were monitored at the same time each day for newly hatched 1st-instar nymphs to quantify the egg period (days). Next, newly-hatched 1st-instar nymphs of *S*. *furcifera* were randomly collected from the NZMF and the GMF treatments, individually transferred to rice seedlings in new numbered glass tubes (one insect per tube), and re-exposed to the NZMF and GMF treatments from which they were collected to complete development. These individuals were checked for molting at the same time daily to monitor development from the 1st-5th instar. The remaining newly-hatched 1st-instar nymphs were reared for the gene expression experiments, and nymphs at 0h, 24h and 60h after molting into the 5th instar from each treatment group meeting the requirements of the molecular experiments were individually transferred into 1.5ml clear microtubes (Axygen MCT-150-C) at and stored in a -80°C freezer (Thermo Scientific Forma 702, USA) (90 individuals for each sampling time).

### Wing dimorphism, fecundity and longevity of female adults

Once the *S*. *furcifera* adults emerged, individuals from each treatment group were identified to sex and wing form. Fifteen pairs of macropterous females and males were randomly selected to mate and oviposit in numbered glass tubes with rice seedlings (Male NZMF x female NZMF and male GMF x Female GMF). Since each *S*. *furcifera* female adult mates multiple times with male adults, a new male adult was added to the cage if the original died before the female. Fecundity was measured by dissecting rice stems every day under a stereomicroscope (MOTIC SMZ-168) until death of the given female individual and counting number of eggs laid per female. Another 40 newly-emerged macropterous females from each treatment group were transferred to large beakers with rice seedlings, maintained continuously under their corresponding magnetic field treatments, and checked for mortality daily until death. The remaining macropterous female adults on the 1st, 4th, 8th day after emergence from each treatment group meeting the requirements of the molecular experiments were individually transferred into 1.5ml clear microtubes (Axygen MCT-150-C) at and stored in a -80°C freezer (Thermo Scientific Forma 702, USA) for the gene expression experiments (90 individuals for each sampling time). Fresh rice seedlings were provided every 3rd day.

### Quantitative real-time polymerase chain reaction (qRT-PCR) analysis of *cryptochromes*, genes in juvenile and ecdysone pathways, and *Vitellogenin*

The duration of the 5th instar, longevity and frequency of wing polymorphism in the *S*. *furcifera* female adults were significantly affected by the NZMF (see Results). For the qRT-PCR analysis of the *cryptochromes* (*CRY1 and CRY2*), *JHAMT*, *FAMeT*, *JHEH* and CYP307A1, RNA was extracted with Trizol (Invitrogen) from newly-molted to 60 hour-old (i.e., 0, 24 and 60 hour-old) 5th instar nymphs, and from newly emerged to 8-day-old (i.e., 1, 4 and 8 day-old) macropterous virgin female adults from both the NZMF and GMF treatment groups. For the qRT-PCR analysis of *Vg*, total RNA was extracted from newly-emerged to 8 day-old (i.e., 1, 4 and 8 days) macropterous virgin female adults from both treatment groups. A pooled sample of 30 heads for each sampling time was randomly mixed as one replicate, and three replicates were analyzed for each sampling interval and magnetic field treatment. Concentration and quality of total RNA was determined using a NanoDrop spectrophotometer (Thermo Scientific) and first-strand complementary cDNA was synthesized using PrimeScript RT reagent kit (TaKaRa). QRT-PCR was performed using SYBR *Premix Ex Taq* (Tli RNaseH Plus) (TaKaRa) in combination with a 7500 Real-Time PCR Detection System. Reactions were performed in a 20μl final volume reaction, using primers in a final concentration of 200nM. Two μl of a 1/2 dilution of the cDNA template of 5th instar nymph and 2μl of a 1/10 dilution of the cDNA template from female adults were used to make the Ct values fall within the suitable range of 15 to 35 based on preliminary runs. No template was added to negative control reactions. *ARF* and *RPL9* were used for housekeeping reference genes for 5th instar nymphs, whereas *18S* and *RPL9* were used for female adults. The gene-specific qRT-PCR primers are listed in Table 1.

**Table 1:** Primers used in the qRT-PCR experiments to measure gene expression levels of *CRY1*, *CRY2*, *JHAMT*, *FAMeT*, *JHEH*, *CYP307A1* in 5th instar nymphs, and *CRY1*, *CRY2*, *JHAMT*, *CYP307A1*, *Vg* in macropterous virgin female adults of *Sogatella furcifera*.

Gene	Description	Primer forward (Sequence 5' to 3')	Primer reverse (Sequence 5' to 3')	GeneBank Accession
*18S*	Housekeeping genes	TGTCTGCTTAATTGCGATAACGAAC	CCTCAAACTTCCATCGGCTTG	JF773150.1
*RPL9*	Housekeeping genes	TGTGTGACCACCGAGAACAACTCA	ACGATGAGCTCGTCCTTCTGCTTT	From Guo-Qing Li
*ARF*	Housekeeping genes	CACAATATCACCGACTTTGGGATTC	CAGATCAGACCGTCCGTACTCTC	From Guo-Qing Li
*CRY1*	Cryptochrome1	CTGTTCTTCCAGCGGCAAC	TGCTCTCACTGCGTCTGTCC	From Guo-Qing Li
*CRY2*	Cryptochrome2	CCTTCTGCTATCACGTTTGCT	CACGCCAAATTATTTCAAGTTCGTC	From Guo-Qing Li
*JHAMT*	JH acid methyltransferase	TGAATTGACTGCCATTACGGTT	CAGTTGTGTTGTTCCCGCTCA	From Guo-Qing Li
*FAMeT*	Farsoic acid methyltransferase	TGAGTATAAGCCTTCAGTACCTAGC	GTTTCACAAGGCATTCCTCTCG	From Guo-Qing Li
*JHEH*	JH epoxide hydrolase	GCACTATAACATCTTCAATGCGACT	AACTCATTTGGGAATCTTGCACA	From Guo-Qing Li
*Vg*	Vitellogenin	CTGATCTGGCTTTCATAGCTCT	GCTGCCAACATGGATCAGAAC	From Guo-Qing Li
*CYP307A1*	Cytochrome P450, family 307, subfamily A	GAGCCCAAAGACTTCACCGAT	CCAGCTCATAAAGAATGTGATGCC	KC701459.1

### Data analysis

All data were analyzed using the SPSS 20.0 (SPSS Inc., Chicago, IL, USA). Significant differences in temperature or RH between the NZMF and GMF treatments during the egg, nymphal, adult and total developmental periods were tested with paired t-test at α = 0.05. The developmental periods of the 1st-5th instar nymphs and total nymph periods were separately analyzed using two-way analyses of variance (ANOVAs), with the magnetic field as main factor (NZMF vs. GMF) and sex (female vs. male) as sub-factor for the macropterous *S*. *furcifera*, and with the magnetic field as main factor (NZMF vs. GMF) and wing form (macropterous female vs. brachypterous female) as sub-factor for the female *S*. *furcifera*. Moreover, two-way ANOVAs were also used to analyze the effects of the magnetic field (NZMF vs. GMF), sampling time, and their interactions on gene expression levels of *CRY1*, *CRY2*, *JHAMT*, *FAMeT*, *JHEH* and *CYP307A1* in the 5th instar nymphs, and on the gene expression levels of *CRY1*, *CRY2*, *JHAMT*, *CYP307A1* and *Vg* in the macropterous virgin female adults. If significant effects of magnetic field, sex/wing form/sampling time or their interactions on the growth, development and reproduction of *S*. *furcifera* were found, follow-up pairwise student's *t* tests were used to compare the means between NZMF and GMF, female and male, or macropterous female and brachypterous female at α = 0.05. Given that vitellin (Vn) of the female is the main source of nutrients for eggs they laid [33], we analyzed the effects of the magnetic field (NZMF vs. GMF) on egg period by one-way ANOVAs, not considering the effect of wing form. One-way ANOVAs were also used to analyze the effects of the magnetic field (NZMF vs. GMF) on longevity and fecundity of macropterous female adults. The Chi-square test was performed to analyze the frequency of brachypterous and macropterous female adults under the NZMF vs. GMF.

## Results

### Effects of the NZMF on the developmental period of eggs laid by macropterous female adults

**Fig 2:**
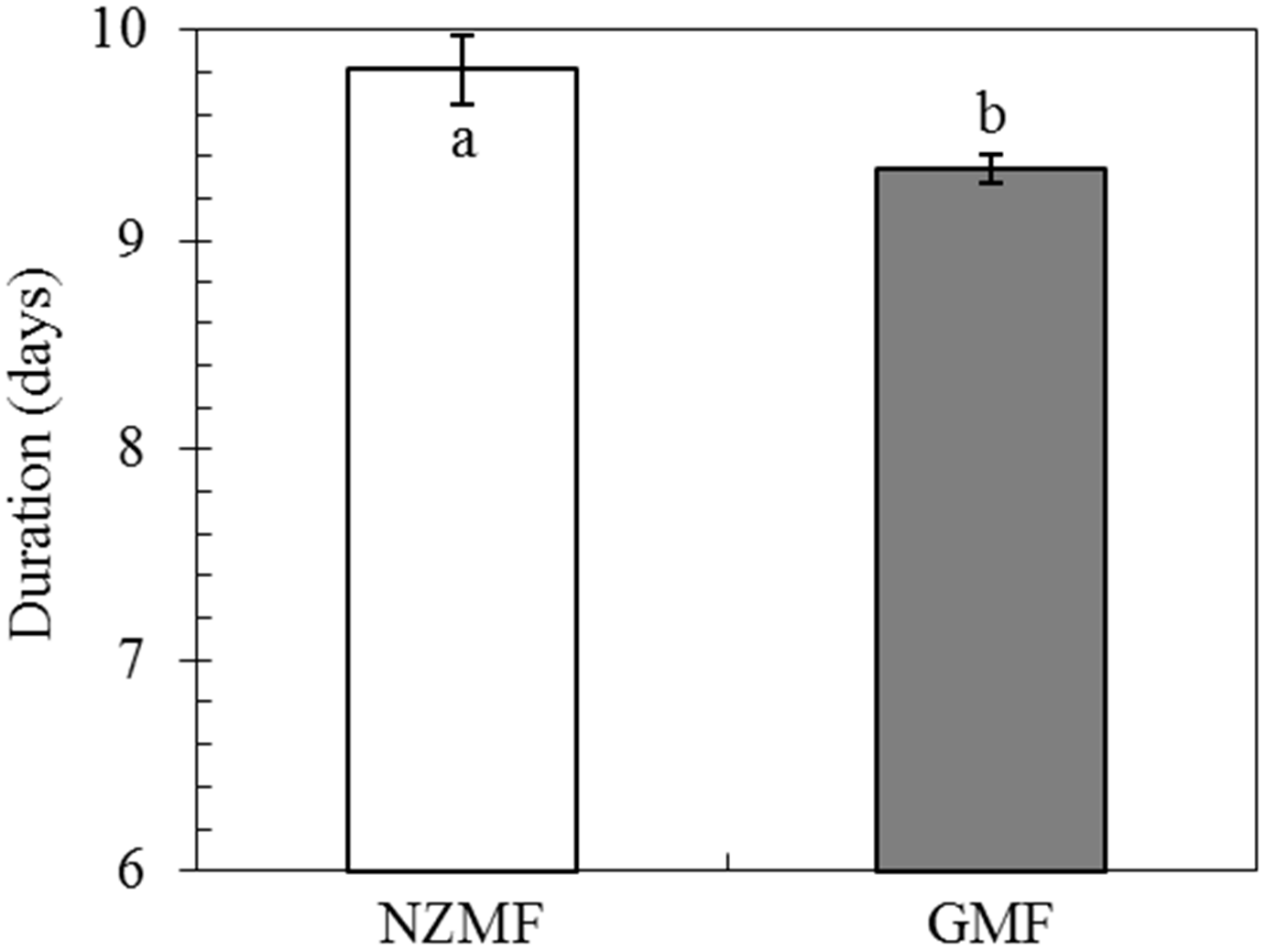
Developmental period of eggs laid by macropterous female adults of *Sogatella furcifera*, under near-zero magnetic field (NZMF) vs. geomagnetic field (GMF). N = 279 and 300 for eggs under the NZMF and GMF, respectively. The columns represent averages with vertical bars indicating SE. Different lowercase letters indicate significant differences between NZMF and GMF treatments for eggs laid by macropterous female adults by one-way ANOVA at *P*<0.05.

Compared with the GMF, NZMF exposure significantly lengthened the developmental period of eggs laid by macropterous female adults by 5.08% on average (*F* = 45.33, *P*<0.001; Fig 2).

### Effects of NZMF on nymphal development

**Fig 3:**
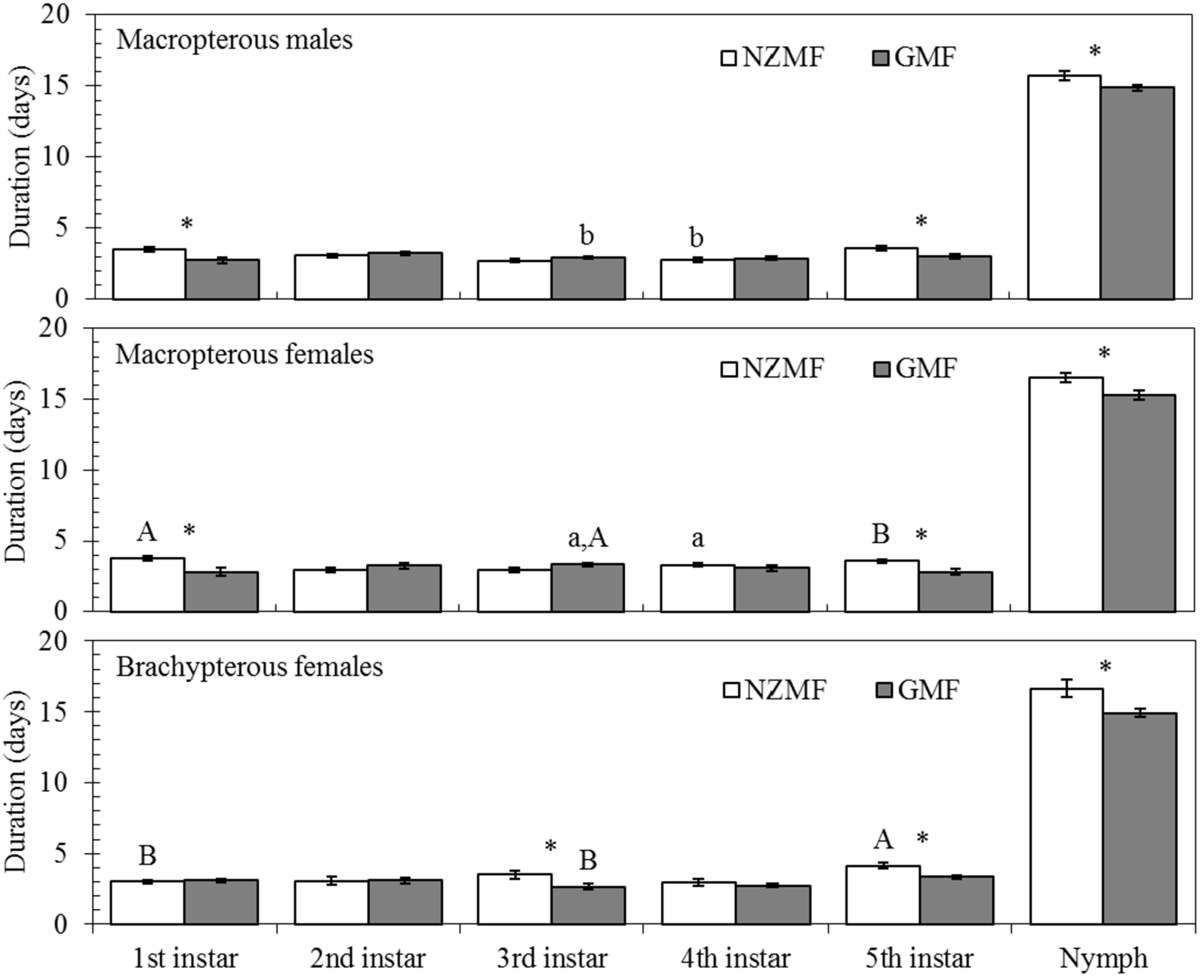
Nymphal periods of macropterous males, macropterous females and brachypterous females of *Sogatella furcifera* under near-zero magnetic field (NZMF) vs. geomagnetic field (GMF). N = 30 for each treatment. The columns represent averages with vertical bars indicating SE. Only significant differences are marked with letters. Different lowercase letters indicate significant differences between macropterous females and males. Different uppercase letters indicate significant differences between macropterous and brachypterous female under the NZMF or GMF by one-way ANOVA at *P*<0.05; * Significant differences between NZMF and GMF for males or females were measured by one-way ANOVA at *P*<0.05.

Across all female and male nymphs, NZMF significantly prolonged the duration of the 1st (*F =* 17.85, *P*<0.001), 5th instar (*F =* 16.98, *P*<0.001) and total nymphal period (*F =* 9.77, *P* = 0.002), and no significant effects of the interaction between magnetic field and sex were found (*F*≤1.01, *P*≥0.32) (Table 2). For the macropterous and brachypterous females, NZMF significantly prolonged the duration of the 1st (*F =* 4.32, *P* = 0.04), 5th instar (*F =* 22.15, *P*<0.001) and total nymphal period (*F =* 9.73, *P* = 0.003) (Table 2). Significant effects of the interaction between magnetic field and wing form were also observed in the 1st (*F =* 6.00, *P* = 0.02) and 3rd instar (*F =* 8.15, *P* = 0.006) (Table 2). In addition, the durations of the 1st, 5th instar and total nymphal stage were significantly prolonged by 27.54%, 19.41% and 5.78% on average for the macropterous male (*P*<0.05; Fig 3), and 33.22%, 26.50% and 7.89% on average for macropterous females (*P*<0.05; Fig 3), respectively. The duration of the 3rd, 5th and total nymphal stage were significantly prolonged by 31.09%, 24.02% and 11.46% on average for the brachypterous females (*P*<0.05; Fig 3). Moreover, significant differences were also found between macropterous males and females in the 3rd instar for the GMF and in the 4th instar for the NZMF treatments (*P*<0.05; Fig 3). Significant differences were also found between macropterous and brachypterous females for NZMF in the 1st and 5th instars, and for GMF in the 3rd instar (*P*<0.05; Fig 3).

**Table 2:** Two-way analyses of variance (ANOVAs) with magnetic field (MF) as main factor and sex/wing form as sub-factor on the duration of the 1st–5th instars and total nymphal periods (*F*/*P*).

Nymph duration	Male & female			Female		
	MF ^a^	Sex	MF ^a^ × Sex	MF ^a^	Wing form ^b^	MF × Wing form ^b^
1st instar	17.85 / <0.001 ***	0.65 / 0.42	0.20 / 0.66	4.32 / 0.04 *	1.62 / 0.21	6.00 / 0.02 *
2nd instar	2.00 / 0.16	0.16 / 0.69	0.21 / 0.65	1.11 / 0.30	0.07 / 0.80	0.11 / 0.74
3rd instar	3.30 / 0.07	3.34 / 0.07	0.22 / 0.64	1.36 / 0.25	0.17 / 0.69	8.15 / 0.006 **
4th instar	0.14 / 0.71	5.08 / 0.03 *	1.01 / 0.32	0.69 / 0.41	2.54 / 0.12	0.12 / 0.73
5th instar	16.98 / <0.001 ***	0.64 / 0.43	0.23 / 0.64	22.15 / <0.001 ***	9.5 / 0.003 **	0.05 / 0.84
Nymph	9.77 / 0.002 **	3.58 / 0.06	0.27 / 0.60	9.73 / 0.003 **	0.04 / 0.89	0.15 / 0.70

^a^ MF—the near-zero magnetic field (NZMF) vs. the geomagnetic field (GMF).

^b^ Wing form—macropterous vs. brachypterous female.

* *P*<0.05.

** *P*<0.01.

*** *P*<0.001.

### Effects of the NZMF on wing dimorphism of female adults

**Fig 4:**
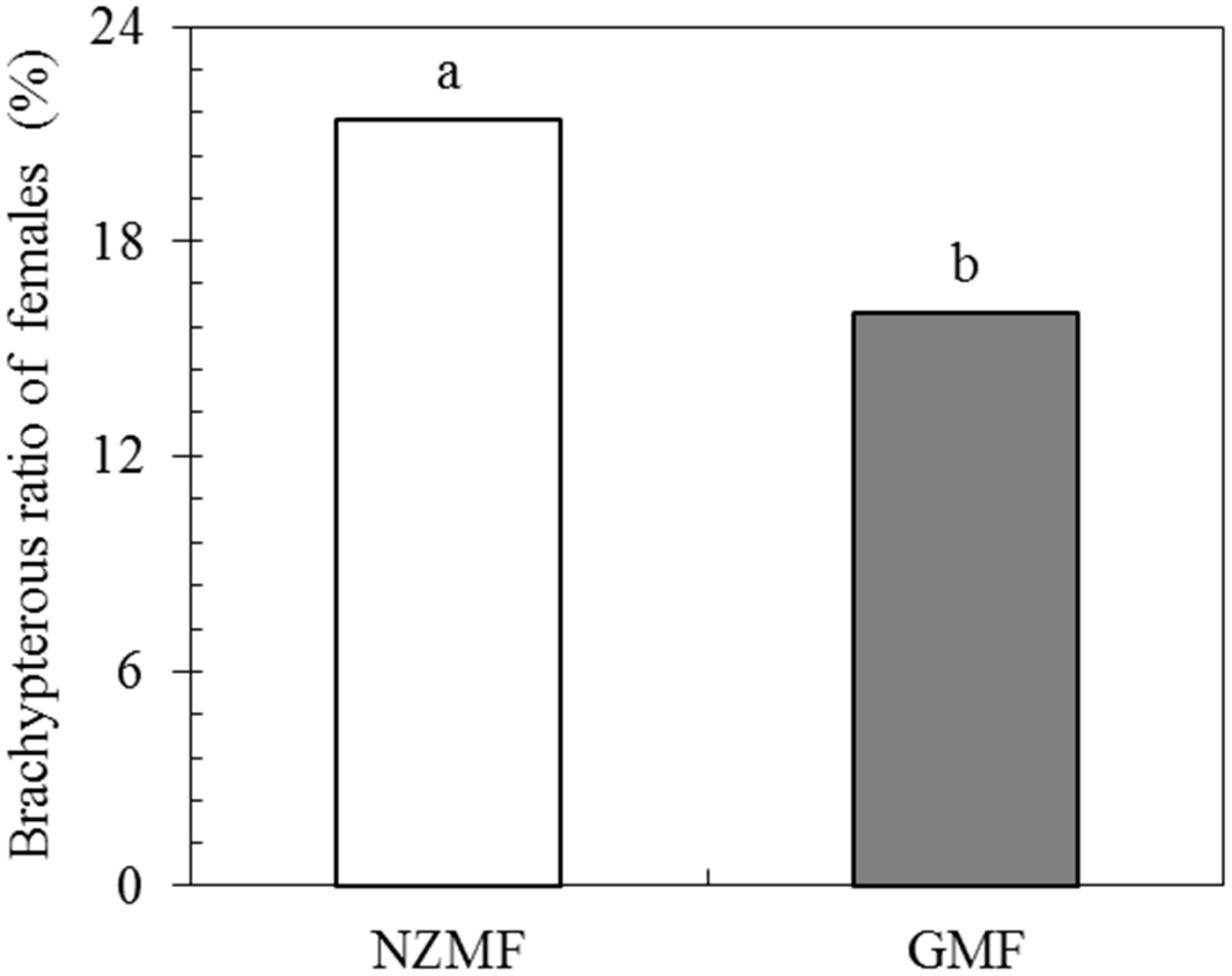
The percentage of brachypterous female adults of *Sogatella furcifera* produced under near-zero magnetic field (NZMF) vs. geomagnetic field (GMF). **U**nder NZMF, 452 and 123 individual newly emerged female adults were macropterous and brachypterous, respectively, whereas under GMF, 342 and 65 females were macropterous and brachypterous, respectively. Insects in both magnetic field treatments came from the same number of parents and were reared for one generation under either NZMF or GMF. Different lowercase letters showed significant differences between NZMF and GMF by the Chi-square test at *P*<0.05.

There was a significant difference in the frequency of brachypterous females produced among insects exposed to NZMF for one generation (*X* ^*2*^ = 4.524, two-tailed *P* = 0.033 by the Chi-square test; Fig 4). Compared with GMF, NZMF significantly increased the frequency of brachypterous female adults by 5.42% (*P* < 0.05; Fig 4).

### Effects of the NZMF on longevity of macropterous virgin female adults

**Fig 5:**
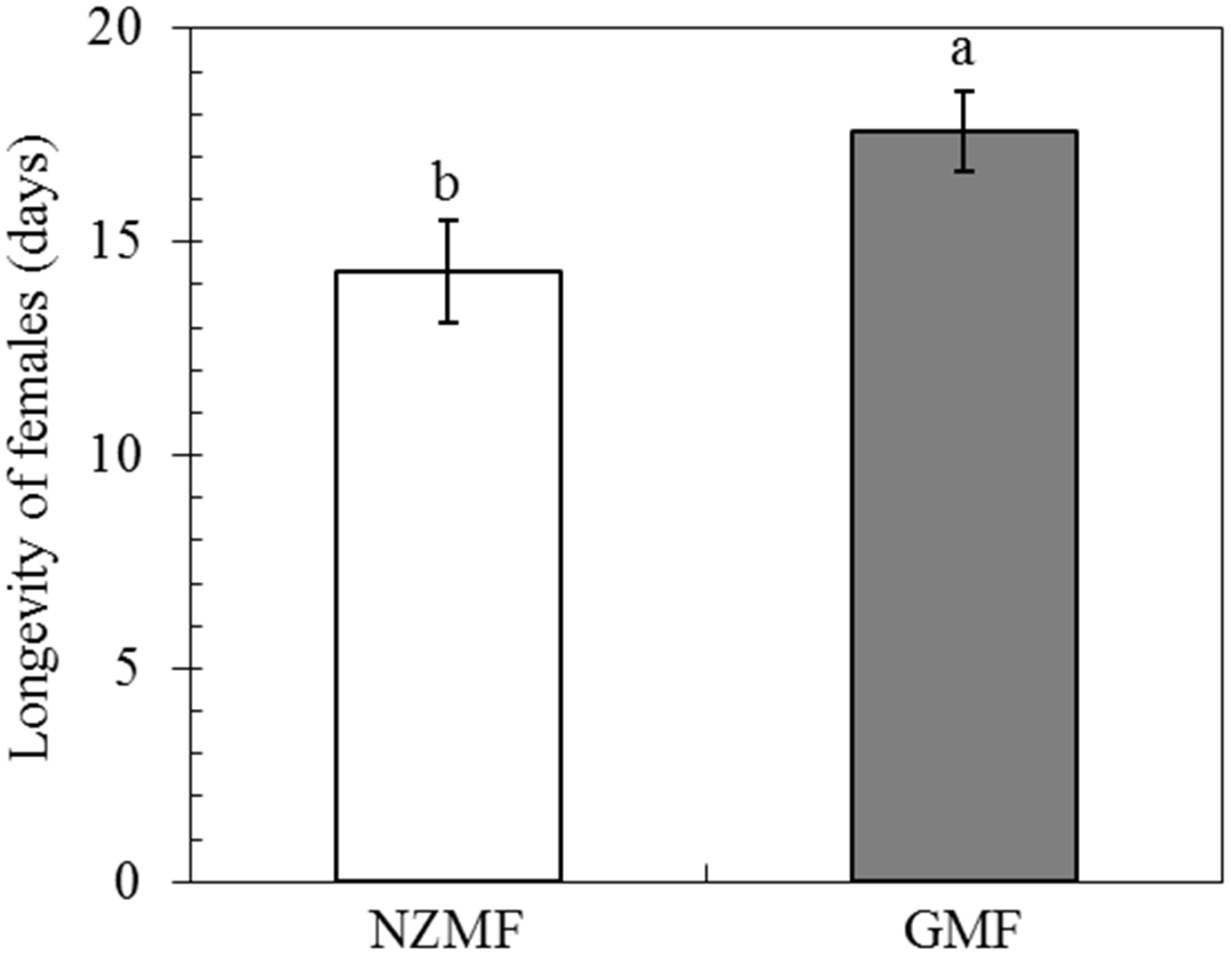
Longevity of macropterous virgin female adults of *Sogatella furcifera*, under near-zero magnetic field (NZMF) vs. geomagnetic field (GMF). N = 30 and 45 for macropterous virgin female adults under the NZMF and GMF, respectively. The columns represent averages with vertical bars indicating SE. Significant differences between NZMF and GMF were measured by one-way ANOVA at *P*<0.05.

Compared with the GMF, the NZMF significantly reduced the longevity of macropterous virgin female adults by 18.67%(*F =* 5.28, *P* = 0.025; Fig 5).

### Effects of the NZMF on fecundity of macropterous female adults

**Fig 6:**
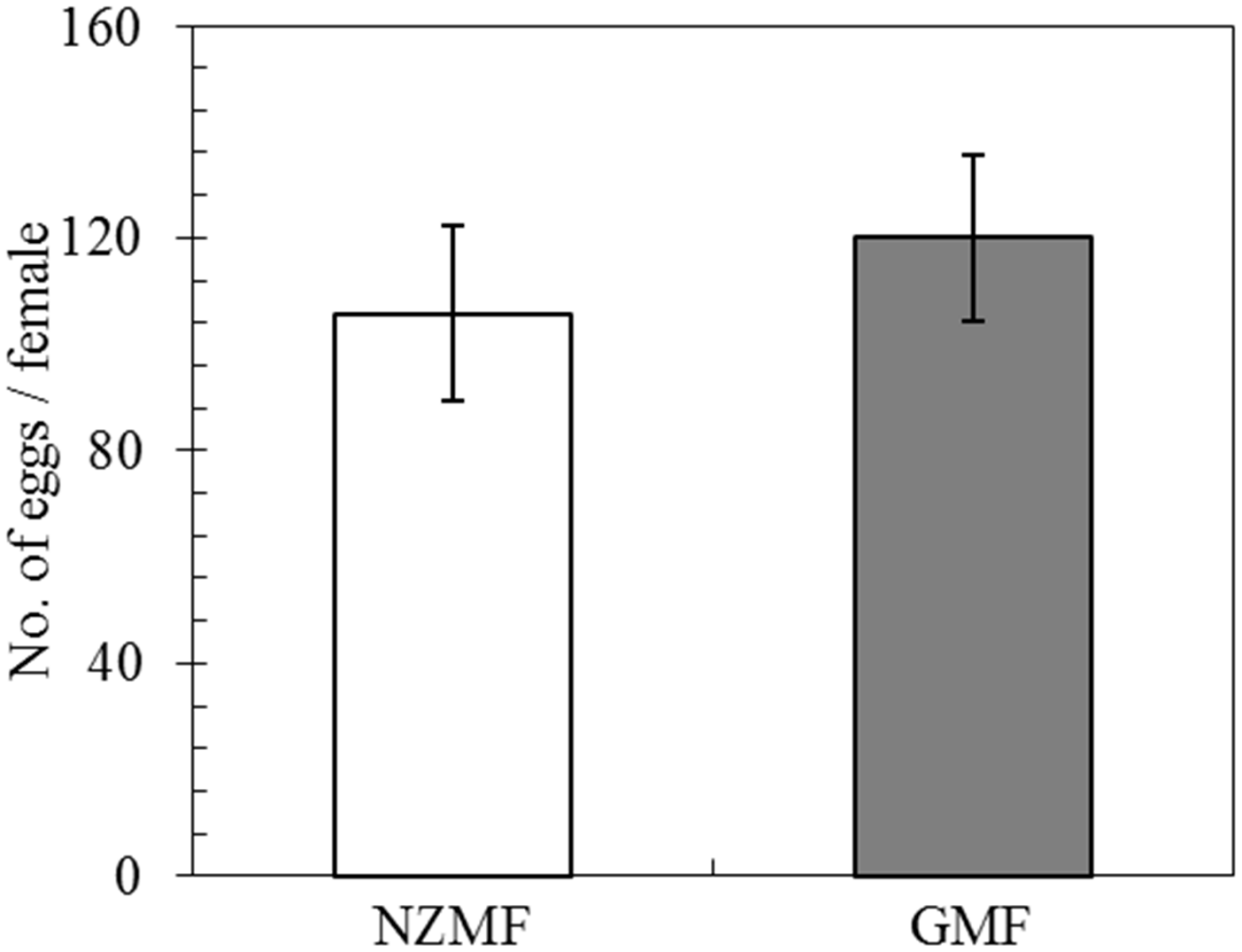
The fecundity of macropterous female adults of *Sogatella furcifera* under near-zero magnetic field (NZMF) vs. geomagnetic field (GMF). N = 15 for both macropterous female adults under NZMF and GMF. The columns represent averages with vertical bars indicating SE. No significant differences between NZMF and GMF were found by one-way ANOVA at *P*<0.05.

NZMF had no significant effects on the fecundity of macropterous female adults mated with macropterous male adults compared to the GMF (*F =* 0.92, *P* = 0.35; Fig 6).

### Temporal expression levels of *cryptochromes* and genes in ecdysone and juvenile hormone (JH) pathways in 5th instar nymphs

**Fig 7:**
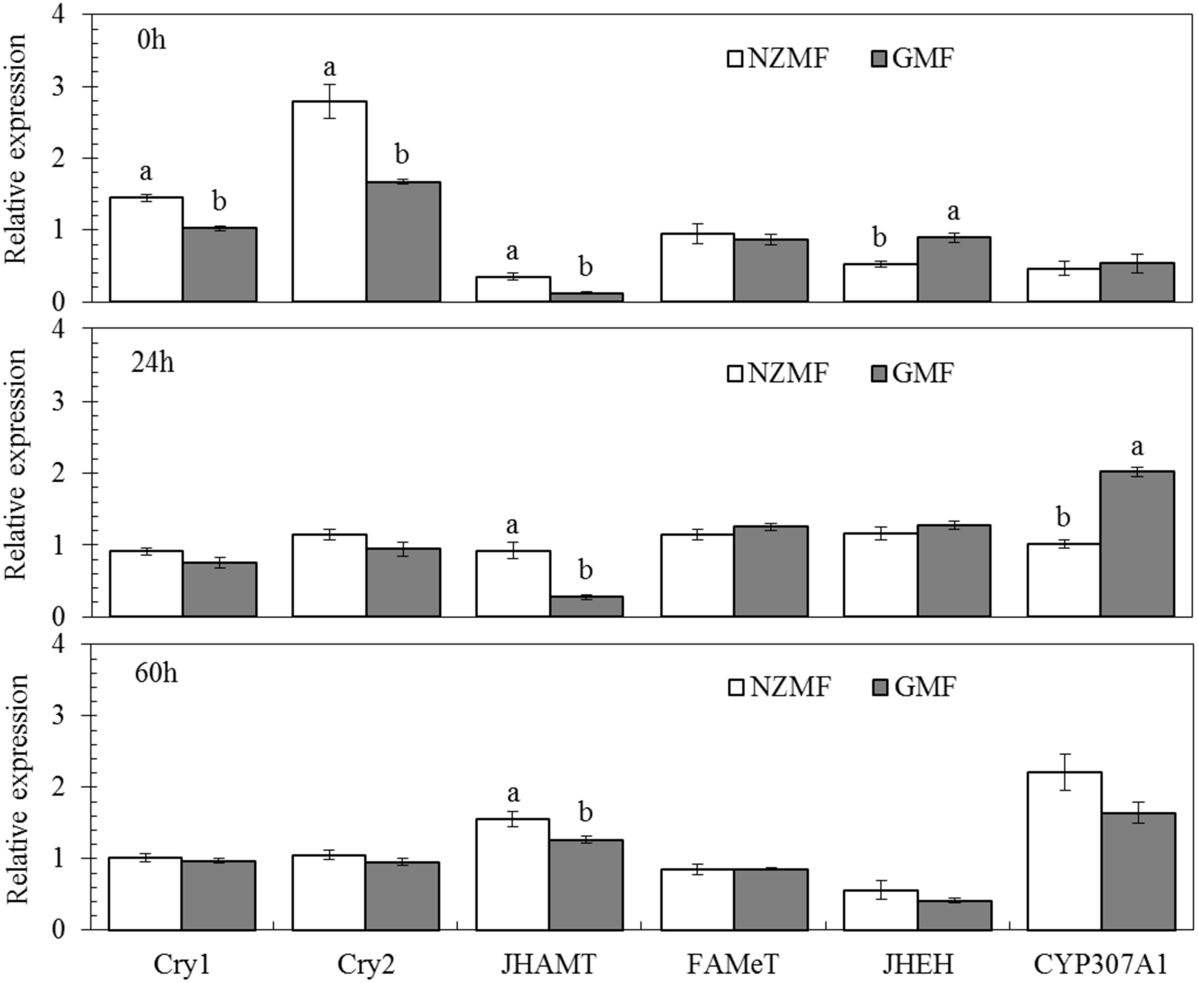
The temporal patterns of gene expression of *cryptochromes* (*CRY1* and *CRY2*), *JHAMT*, *FAMeT*, *JHEH* and *CYP307A1* at 0h, 24h and 60h after molting into the 5th instar of *Sogatella furcifera*, under near-zero magnetic field (NZMF) vs. geomagnetic field (GMF). Thirty individual 5th instar nymphs were randomly mixed as one sample for each sampling time with three repeats. The columns represent averages with vertical bars indicating SE. Only significant differences are marked with letters. Different lowercase letters indicate significant differences between NZMF and GMF for the same sampling time by the Student's *t*-test at *P*<0.05.

The expression of the *cryptochrome* genes (*CRY1* and *CRY2*), *CYP307A1*, *JHAMT*, *FAMeT* and *JHEH* in 5th instar insects at 0h, 24h and 60h after molting varied significantly over time (*F*≥11.13, *P*≤0.002; Table 3). Magnetic field significantly affected the expression of *JHAMT* and both *cryptochromes* (*CRY1* and *CRY2*) (*F*≥25.03, *P*≤0.001; Table 3). The temporal patterns of expression varied for the different genes under different magnetic field treatments as evidenced by significant interactions between magnetic field and sampling time for *CRY1*, *CRY2*, *CYP307A1*, *JHAMT* and *JHEH* (*F*≥5.64, *P*≤0.019; Table 3). Interestingly, the *cryptochromes* (*CRY1* and *CRY2*) were significantly up-regulated by NZMF vs. GMF at 0h after molting into the 5th instar (*P*<0.05; Fig 7). Compared with the GMF, NZMF significantly increased *JHAMT* expression levels at the 0h, 24h and 60h after molting into the 5th instar (*P*<0.05). Simultaneously, NZMF reduced the *JHEH* expression level at the 0h after molting into the 5th instar (*P*<0.05; Fig 7). *CYP307A1* exhibited significantly lower expression at 24h (*P*<0.05), but higher expression levels at the 60h after molting into the 5th instar nymphs under NZMF vs. GMF (*P*>0.05; Fig 7). No significant differences were found in the *FAMeT* expression levels between the NZMF and the GMF at any time during the 5th instar (*P*>0.05; Fig 7).

**Table 3:** Two-way ANOVAs with magnetic field (MF) as main factor and sampling time as sub-factor on the gene expression levels of *cryptochromes* (*CRY1* and *CRY2*), *JHAMT*, *FAMeT*, *JHEH* and *CYP307A1* for 5th instar nymphs (*F*/*P*).

Genes	MF ^a^	Sampling time ^b^	MF ^a^ × Sampling time ^b^
*CRY1*	26.49 / <0.001 ***	33.21 / <0.001 ***	7.74 / 0.007 **
*CRY2*	25.03 / <0.001 ***	73.28 / <0.001 ***	11.86 / 0.001 **
*JHAMT*	71.37 / <0.001 ***	145.38 / <0.001 ***	13.64 / 0.001 **
*FAMeT*	0.03 / 0.87	11.13 / 0.002 **	0.77 / 0.485
*JHEH*	3.39 / 0.09	47.70 / <0.001 ***	5.64 / 0.019 *
*CYP307A1*	2.11 / 0.17	54.06 / <0.001 ***	15.70 / <0.001 ***

^a^ MF—the near-zero magnetic field (NZMF) vs. the geomagnetic field (GMF).

^b^ Sampling time—0h, 24h and 60h after molting into the 5th instar under NZMF and GMF.

* *P*<0.05.

** *P*<0.01.

*** *P*<0.001.

### Temporal expression levels of *Vg*, *cryptochromes* and genes in ecdysone and juvenile hormone (JH) pathways in macropterous virgin female adults

**Fig 8:**
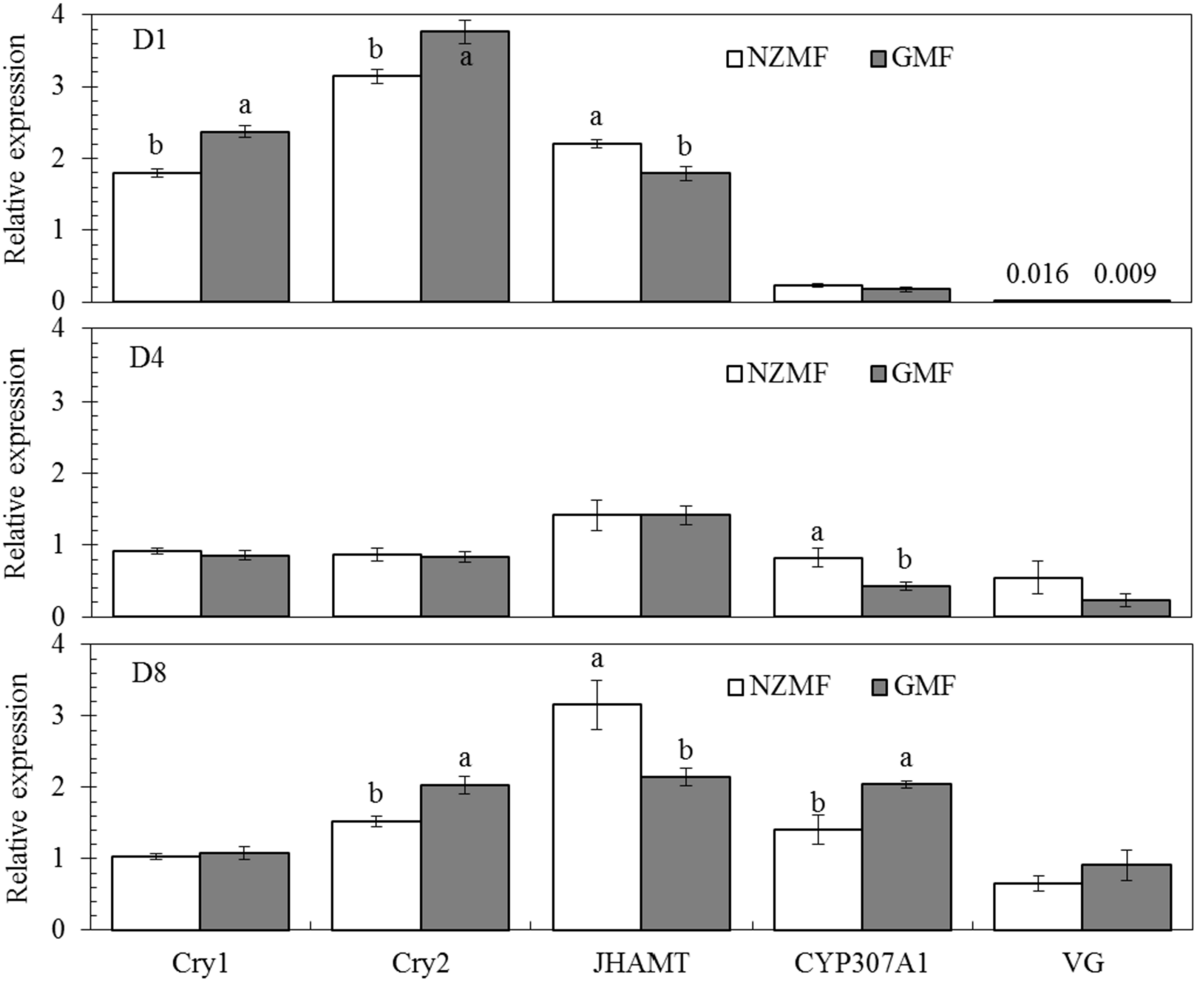
Temporal gene expression patterns of *cryptochromes* (*CRY1* and *CRY2*), *JHAMT*, *CYP307A1* and *Vg* on the 1st (D1), 4th (D4), 8th (D8) day after emergence of macropterous female adult *Sogatella furcifera* under near-zero magnetic field (NZMF) vs. the geomagnetic field (GMF). Thirty individual macropterous female adults were randomly mixed as one sample for each sampling time with three repeats. The columns represent averages with vertical bars indicating SE. Only significant differences are marked with letters. Different lowercase letters indicate significant differences between NZMF and GMF for the same sampling time by the Student's *t*-test at *P*<0.05.

The two-way ANOVAs indicated that NZMF significantly affected the expression of *CRY1*, *CRY2* and *JHAMT* in macropterous virgin female adults (*F*≥9.90, *P*≤0.008; Table 4). Significant effects of sampling time (*F*≥11.13, *P*≤0.002; Table 4) were found for all five of the genes assayed. Moreover, significant magnetic field × sampling time interactions for the *cryptochromes* (*CRY1* and *CRY2*) and *CYP307A1* (*F*≥5.21, *P*≤0.02; Table 4) indicated that the expression patterns of these genes differed over time. Compared with the GMF, NZMF significantly reduced the expression levels of *CRY1* on the 1st day, *CRY2* on the 1st, 8th day and *CYP307A1* on the 8th day after emergence of macropterous virgin female adults (*P*<0.05; Fig 8), while the expression levels of *JHAMT* on the 1st, 8th day, and *CYP307A1* on the 4th day after emergence of macropterous virgin female adults were significantly up-regulated by the NZMF (*P*<0.05; Fig 8).

**Table 4:** Two-way ANOVAs with magnetic field (MF) as main factor, sampling time as sub-factor on the gene expression of *cryptochromes* (*CRY1* and *CRY2*), *JHAMT*, *CYP307A1* and *Vg* for the macropterous virgin female adults (*F*/*P*).

Genes	MF ^a^	Sampling time ^b^	MF ^a^ × Sampling time ^b^
*CRY1*	12.14 / 0.005 **	194.71 / <0.001 ***	13.16 / 0.001 **
*CRY2*	16.87 / 0.001 **	293.52 / <0.001 ***	5.21 / 0.02 *
*JHAMT*	9.90 / 0.008 **	22.06 / <0.001 ***	3.83 / 0.052
*CYP307A1*	0.55 / 0.47	115.56 / <0.001 ***	12.90 / 0.001**
*Vg*	0.03 / 0.86	14.72 / 0.001 **	2.03 / 0.174

^a^ MF—the near-zero magnetic field (NZMF) vs. the geomagnetic field (GMF).

^b^ Sampling time—the 0, 4 and 8 days after emergence of female adult and the GMF.

* *P*<0.05.

** *P*<0.01.

*** *P*<0.001.

## Discussion

We have demonstrated a series of significant MFE induced by exposure to NZMF on the development and physiology of *S*. *furcifera* ranging from variation in gene transcription to the expression of alternative phenotypes. Combined with the results of our previous work using the same approach [2], this study broadens our understanding of NZMF triggered MFE on *S*. *furcifera* development to include variation depending on species, sex and alternative wing-form phenotype.

It is well-known that the main ecdysone, 20E and JH coordinately orchestrate larva to larva (or nymph to nymph) and metamorphosis development [14–18]. Positive correlations have been revealed between *JHAMT* expression levels and JH titer as well as between *CYP307A1* expression levels and 20E titer. It was also reported that one major route of insect JH degradation is epoxide hydration by JH epoxide hydrolase (JHEH). Given these relationships, our findings in gene expression levels of *JHAMT*, *JHEH* and *CYP307A1* in 5th instar nymphs are consistent with an up-regulation of JH and a down-regulation of 20E. Therefore, change patterns of these two hormones in *S*. *furcifera* should be related to the significantly prolonged nymphal development we observed in this and the previous study [2]. Besides the roles of JH in juvenile development, a role for JH in regulating insect dormancy was recently shown in experiments in which surgical removal of the CA led to reduced fecundity or sterility and extended longevity in grasshoppers, butterflies, hemipterans, and fruit flies, and it was suggested that JH is a positive regulator of fecundity, but a negative regulator of life span [35]. It was also reported that JH is involved in the regulation of wing dimorphism and the development of brachypterous wings in *N*. *lugens* [36]. Our findings in the related *S*. *furcifera* demonstrated changes in gene expression levels of *JHAMT*, *JHEH* in 5th instar nymphs and *JHAMT* in macropterous virgin female adults that are consistent with an up-regulation of JH, which should be responsible for the significantly higher brachypterous forms and significantly shorter life span of macropterous virgin female adults under the NZMF compared to the GMF. Although hormone levels in the egg stage were not determined in our study, the prolonged hatching period we observed was likely to be triggered by hormone signal transduction as well [37]. All insects provision their eggs with Vn as a major yolk protein, and Vg acts as the precursor of Vn. Vg molecules in the fat body are important in facilitating the transport of nutrients to the ovaries [33]. Zhai *et al*. (2013) have shown that the number of brown planthopper offspring decreased after its nymphs (third to fifth instars) were continuously treated with ds*Vg* [34], which indicated a positive correlation between gene expression level of *Vg* and fecundity. This positive correlation may be the reason why significant differences in fecundity were not found between GMF and NZMF treated macropterous adult females (Fig 6). However, in comparison with the temporal gene expression pattern of *Vg* under the GMF in our study, it seemed that the transcription of *Vg* commenced earlier when exposed to the NZMF. Given that the hormones involved in *Vg* gene regulation include juvenile hormone, ecdysone and several neuropeptides [32], our observation of alternating changes between *JHAMT* and *CYP307A1* may have been responsible for the gene expression pattern of *Vg* in female adults, though with a time lag.

In a recent transcriptome profiling analysis of human neuroblastoma cells, *MAPK1* and *CRY2* showed significant up- and down-regulation, respectively, during the first 6 h of a NZMF exposure [38]. We also found significant changes in the expression of both *CRY1 and CRY2* in 5th instar and adult females of *S*. *furcifera*. With the exception of the down-regulation of *CRY2* on day 8 in macropterous females, significant changes in both life history stages were only early in the developmental period, implying that *cryptochromes* may be located upstream in magnetic response as putative magnetosensors [9]. Cryptochrome proteins precisely act as components of the central circadian clock of metazoans [39] that times a range of important behavioral and physiological processes in animals including sleep-wake cycles [40], locomotion patterns [41], feeding [42], mating [43], and cell division [44]. Interestingly, NZMF has been reported to affect circadian rhythm [20, 21]. Circadian clock is also found to be altered in space where is absence of electromagnetic field [31, 32]. The work of Yoshii *et al*. (2009) further reveals that the magnetosensitivity of *Drosophila’s* circadian clock need light activation of cryptochrome and depend on the applied field strength [45]. In insects, levels of JH and ecdysone are controlled by the neuroendocrine system [46–50]. Many experiments have shown relationships between the circadian clock and neuroendocrine hormones that may indirectly regulate secretion of JH and ecdysone (e.g. 20E) [14, 18, 50–53]. Most likely, the pacemaker neurons that produce the principal circadian transmitter called pigment-dispersing factor (PDF) may connect the circadian clock to hormone signal transduction. This is supported by the fact that axons projecting from the PDF-producing neurons terminate in close proximity to the dendritic arbors of the prothoracicotropic hormone-producing neurons [17], and PDF mutants show altered prothoracicotropic hormone transcript levels [18] which is reported negatively correlated with JH while positively correlated with ecdysone [15, 18]. In addition, mammalian-type cryptochromes in insects such as the monarch butterfly seem to work as a transcriptional repressor in the feedback loop of the circadian clock [54]. Therefore, the MFE involved in hormone signal transduction here may due to potential roles for magnetic fields in the regulation of circadian clock [5, 45].

**Fig 9:**
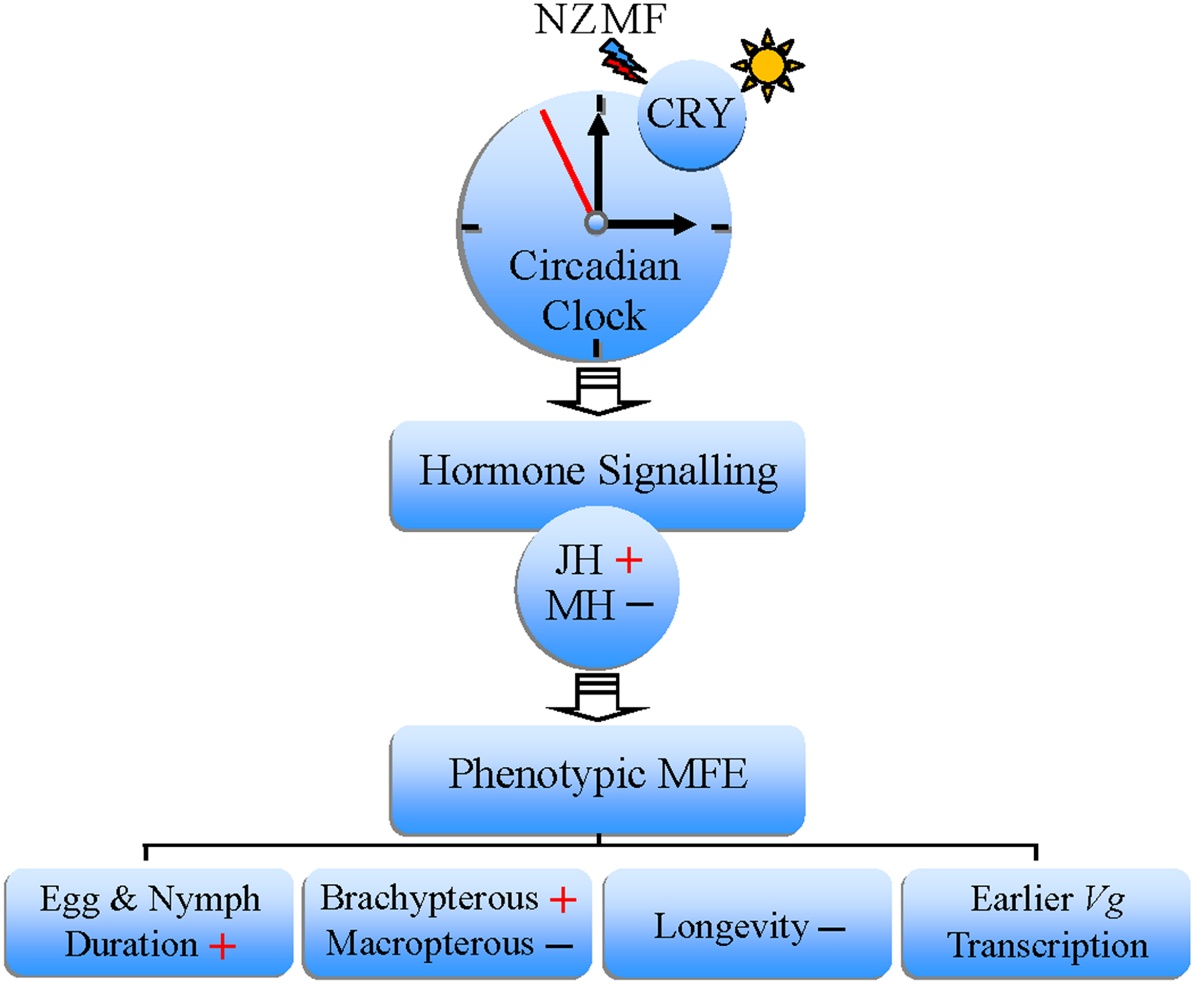
A conceptual model of hormone signal transduction-involved magnetic field effects (MFE), triggered by the near-zero magnetic field (NZMF) on the development and physiology of *Sogatella furcifera*. Abbreviation, CRY: Cryptochrome; JH: Juvenile hormone; MH: Molting hormone (Ecdysone); *Vg*: *vitellogenin*; +: up-regulated or increased;–: down-regulated or decreased.

It is commonly known that continuous treatment with abnormal or unexpected signals would result in stress reactions. Observations with mole-rats suggested that magnetosensitivity is no different to the other senses, and is involved in multi-sensory integration with other inputs [55, 56]. Presumably, the MFE in our study may also be stress responses of sensory systems [56], induced by continuous treatment with the manipulative NZMF. Based on some previous research, our current results, and the published evidence for the putative role of cryptochromes as magnetosensors [6, 9, 12, 13, 45, 57, 58], a conceptual model describing potential links among MFE on the development and physiology of *S*. *furcifera*, hormone signal transduction and magnetosensitivity is provided in Fig 9. Here we proposed that one pathway could be specifically originated from the putative light-dependent magnetosensor, cryptochrome. The effects of the NZMF on the development and physiology of *S*. *furcifera* may be owing to the dual function of cryptochromes, that there may be interactions between their putative function as magnetosensors and as crucial components of the circadian clock [10]. Furthermore, given the above discussion of the potential roles for magnetic fields in the regulation of circadian clock, our results indicating temporal changes induced by NZMF exposure in the expression patterns of *cryptochromes* and genes in juvenile hormone (JH) and ecdysteroid pathway provide a feasible causal link among magnetosensitivity, hormone signal transduction and the phenotypic MFE.

Except for the functional links as light-dependent CRY-mediate pathway in Fig 9, it is also possible that the MFE on cryptochromes, hormone signal transduction and circadian clock of *S*. *furcifera* are isolated and non-functional since the precise biophysical origin of magnetosensitivity or MFE remains unclear. Although the phenotypic MFE of prolonged egg and nymph duration, earlier transcription of *Vg*, decreased longevity of macropterous female adults and increased brachypterous ratio of female adults in *S*. *furcifera* occurred when exposed to the NZMF, there still have much possibilities to be further studied in the future. For example, whether changes in development and physiology under NZMF is also related to altered circadian rhythms of cell division [44], cell proliferation [59], apoptosis [60] and efficiency of food digestion [23], the level of locomotor activity [5] or nutrient efficiency [61]. In summary, our findings of MFE triggered by the NZMF on transcriptional levels of *cryptochromes* and other genes in growth and development-related hormone pathways suggest the presence of a hormone signal transduction mechanism in *S*. *furcifera*. Importantly, such a pathway is conventionally suggested to coexist with neural signal transduction [13], together making up the signal transduction systems of MFE. To our knowledge, this study provides the first molecular evidence that insect hormone signal transduction can contribute to MFE. The use of manipulative NZMF in this study also provides valuable references for exploring potential bioeffects triggered by decrease of the GMF intensity [27, 30] and bioeffects on astronauts in space where is absence of magnetic field [30–32].
